# The Pollinating Network of Pollinators and the Service Value of Pollination in Hanzhong City, China

**DOI:** 10.3390/insects16121223

**Published:** 2025-11-30

**Authors:** Xuemei Chang, Xiaofeng Yan, Fengming Lv, Ying Zhang, Tom D. Breeze, Xiushan Li

**Affiliations:** 1 Key Laboratory of Southwest China Wildlife Resources Conservation of the Ministry of Education, College of Life Science, China West Normal University, Nanchong 637009, China; 2 College of International Cooperation, China West Normal University, Nanchong 637009, China; xiaofeng2425@126.com; 3 College of Forestry Economics and Management, Beijing Forestry University, Beijing 100035, China; zhangyin@bjfu.edu.cn; 4 Centre for Agri-Environmental Research, School of Agriculture, Policy & Development, University of Reading, Reading RG6 6AR, UK; t.d.breeze@reading.ac.uk

**Keywords:** pollinating insect, habitat types, pollination crops, interaction network, pollination service value, protection countermeasures

## Abstract

Pollinating insects are the most important pollinators in nature; they pollinate vegetables, fruits, oil crops, and wild plants, so that crop yields can be increased, wild plants can live and reproduce, and human food security and ecosystem stability are maintained. To identify the pollination network of plants–insects and the pollination service value in Hanzhong City, the methods of random net capture and transect counting in field work were used. The agricultural statistical data from Hanzhong City in 2023 was combined in the analysis. The results showed that Hanzhong City is rich in pollinator resources, with a total of 80 species of pollinators and 59 species of pollinating crops and wild nectar plants. The abundant pollinator resources provide sufficient pollination services for the production of local vegetables, fruits, and oil crops. The characteristics of the pollination networks are obvious, showing the structural characteristics of low connectivity, medium nesting, and low network specialization. In 2023, the pollination service value of pollinators in Hanzhong City was CNY 3524–4878 billion, accounting for 10.02–13.87% of the city’s agricultural output value of the year. Suggestions for the protection of pollinators in Hanzhong City: Reduce the use of pesticide, support beekeeping, intercrop nectar plants, and rationally plant crops.

## Simple Summary

This study investigated the pollinator resources, the pollination network of plants–insects, and the pollination service value in Hanzhong City, using random net capture and transect counting in the field. The results showed that Hanzhong City is rich in pollinator resources, with a total of 80 species of pollinators and 59 species of pollinating crops and wild nectar plants. The abundant pollinator resources provide sufficient pollination services for the production of local vegetables, fruits, and oil crops. The characteristics of the pollination networks are obvious, showing the structural characteristics of low connectivity, medium nesting, and low network specialization. In 2023, the pollination service value of pollinators in Hanzhong City was CNY 3524–4878 billion, accounting for 10.02–13.87% of the city’s agricultural output value of the year. This is higher than the global average. Suggestions for the protection of pollinators in Hanzhong City: Reduce the use of pesticide, support beekeeping, intercrop nectar plants, and rationally plant crops.

## 1. Introduction

Pollinators play an important role in ecosystems by transferring pollen between the male and female parts of flowers, which enables fertilization and reproduction. Pollinators provide numerous benefits to humans, such as the reproduction of a diverse seed and fruit supply, sustaining populations of wild plants that underpin biodiversity and ecosystem function, producing honey and other beekeeping products, and supporting cultural values. The majority of global cultivated and wild plants depend on pollination by animals [1,2].

Much of the recent international focus on pollination services has been on the benefits to food production, as animal pollination directly affects the yield and/or quality of approximately 75% of globally important crop types [3]. These include most fruits, seeds, and nuts, and several high-value commodity crops such as coffee, cocoa, and oilseed rape. An estimated 5–8% of global crop production would be lost without pollination services, resulting in billions of dollars of lost production, which would necessitate changes in human diets and a disproportionate expansion of agricultural land to fill the shortfall in crop production by volume [1,2]. Although a wide range of animals, such as flies, thrips, beetles, birds, and bats, provide pollination, bees are the most important group of pollinators to global crop production, visiting more than 90% of the leading 107 global crop types [1,2].

As a country with great crop and animal biodiversity, China ranks first in the world in the value of pollination services, with a total value of CNY 6790.3 billion in 2015, much of which is exported into global markets [4,5]. China’s wild pollinator fauna is approximately three times as diverse as Europe, with 1342 species of bees [6], 125 species of bumblebees [7] (accounting for 50% of global species), and more than 1700 species of butterflies [8,9]. Within China, pollinator-dependent crops are often very important at a regional level: for example, it is estimated that the value of pollination services in the Nanchong City area accounted for at least 12.88% of local agricultural output value in 2023 [10].

The Hanzhong Plain is located between the Qinling Mountains and the Daba Mountains. The mountainous, hills and plains have formed a diversified agricultural habitat, which has the highest crop production area in Shaanxi Province. Among the crops planted in the plain, *Brassica napus*, *Oryza sativa*, and *Gastrodia elata* are the main crops in the intensive arable systems. As both a national key ecological function area and an agricultural green development pilot area, the Qinling and Daba Mountains are a key source of pollinator natural capital for Hanzhong City and are incorporated into the construction of ecological agriculture demonstration areas. These demonstration areas are established through the collaboration of the private sector and government departments to improve people’s understanding of the pollination function of pollinating insects and their economic contribution to local agricultural production, and to better protect and utilize pollinating insect resources to maintain a healthy, stable ecosystem that promotes local economic development.

Despite the value of pollinators, intensive agricultural practices remain common in much of the world, and there is growing evidence of economically significant pollination service deficits in several parts of the world [11,12]. Such intensive agricultural practices are common in the Hanzhong Plain, and there is little evidence of changes to such practices. Although managed honeybees could theoretically replace the pollination services of wild bees, managed honeybee stocks in China are below the numbers required [13], and beekeeping in China is increasingly sedentary, meaning few beekeepers would be able to provide such services [14]. Understanding the value of pollinators is thought to be crucial to the development of effective management strategies, but these need to be tailored to a local scale [15] and to the needs of key local pollinators [16]. To date, there has been no assessment of the important plant–pollinator networks that could support pollination services in the Hanzhong Plain.

Here, we attempt to address these gaps by exploring the pollinating insect resources in the Hanzhong area, the impact of different habitat types on pollinating insects, and the value of pollination services; in this study, from March to September 2023, the species of pollinator resources were investigated in different habitat types, such as forest, agroforestry ecotone, and agricultural area in Hanzhong City, by means of random collection, transect counting, sampling and fixed-point observation. The influence of habitat types on pollinators was evaluated, and the insect–crop pollination network was constructed. The value of insect pollination service was evaluated, and suggestions for the protection and utilization of local pollinators were put forward.

## 2. Materials and Methods

### 2.1. Study Location

**Figure 1:**
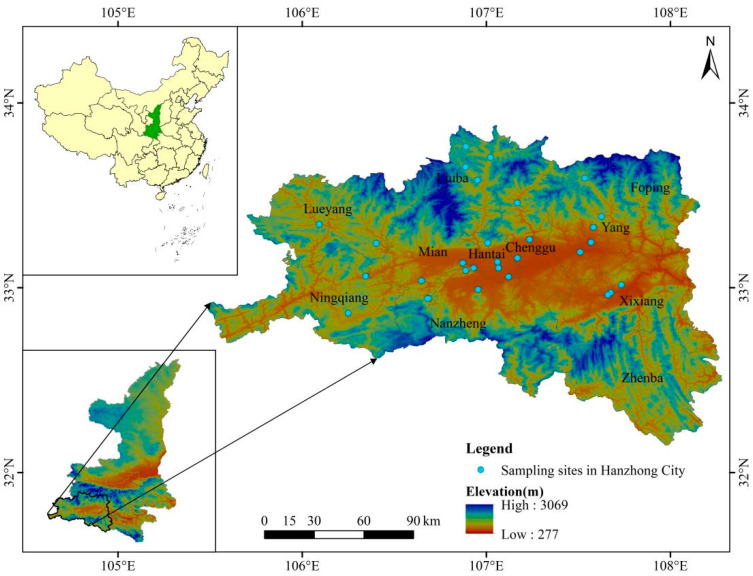
Transect distribution includes 5 transects located in forested areas, 6 in agroforestry ecotones, and 6 in farmland in Hanzhong City Shaanxi Province, China.

The study site is located in the plain agricultural area within the jurisdiction of Hanzhong City, Shaanxi Province, China and in the agroforestry ecotone on the southern slope of the Qinling Mountains and the northern slope of the Daba Mountains. The east longitude is 105°30′–108°24′, and the north latitude is 32°15′–33°56′ (Figure 1).

The climate is subtropical with an average annual temperature of 13.8 °C and a precipitation of 900 mm. The warm and humid climate provides a suitable place for pollinators to survive. The vertical distribution of landforms at altitudes of 1000–2000 m is obvious, creating multiple habitats, such as forests, farmland, and wetlands, and breeding abundant pollinating insects: butterflies, bees, bumblebees, hoverflies, and moths. The rape and other insect-pollinated crops planted in Hanzhong City are continuously distributed, forming a stable food source [17].

### 2.2. Species Survey

A combination of random sampling, observation records, and transect surveys was employed to obtain data on the species, distribution, and pollination crop records of pollinators.

### 2.3. Transect Selection

**Figure 2:**
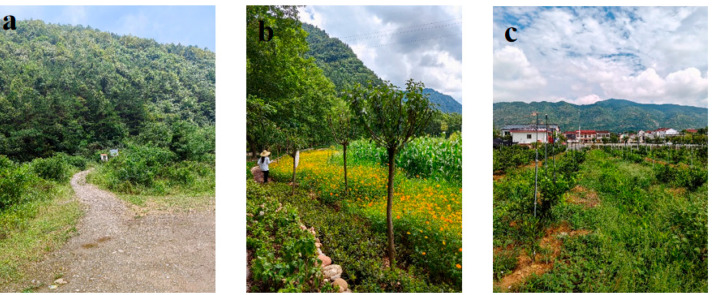
Habitat types in Hanzhong City ((**a**): forest region; (**b**): agroforestry ecotone; (**c**): farmland).

Transects were selected within the study area based on factors such as elevation, terrain, vegetation, and butterfly activity, ranging across three habitat types: forested areas, agroforestry ecotone, and farmland. A total of 17 transects were chosen, each measuring 200 m in length and 5 m in width. Among these, 5 transects were located in forested areas, 6 in agroforestry ecotone, and 6 in farmland. The distribution of transects is shown in Figure 1, while the three habitat types are illustrated in Figure 2.

### 2.4. Transect Survey

The Pollard transect survey method was employed to conduct the survey a total of 6 times at intervals of 20 days in Hanzhong City from March to September 2023, primarily focusing on the diversity of flowering plants and pollinating insects. Each transect was 200 m in length and 5 m in width. Surveys were conducted on sunny, windless days with temperatures above 16 °C. Walking along the selected transect at a speed of 2.5 km/h, insects such as butterflies and bees were collected and recorded within a 2.5 m wide area on both sides, at a height of 5.0 m. Identified species were recorded by habitat type and quantity, while unidentified species were collected using net trapping. Butterflies were stored in triangular paper bags, and bees and hoverflies were collected and immersed in ethanol solution in a 10 mL centrifuge tube, and then brought back to the laboratory for specimen preparation and species identification.

### 2.5. Species Identification

The species were identified based on Chinese Butterfly Monography [8], the Chinese Butterfly Atlas [9], and the Tibetan Bee Atlas of the Second Comprehensive Scientific Survey of the Qinghai–Tibet Plateau [18].

### 2.6. Diversity Index Analysis

The species richness and abundance of pollination were counted, and the Shannon–Wiener diversity index (*H′*), Berger–Parker dominance index (*D′*), and Pielou evenness index *(J*) were calculated in Past 4.12 and Excel 2019.

*H′* evaluates diversity by calculating the relative abundance of each species. The higher the value, the richer the species.

*D′* ranges between 0 and 1; the higher the value, the more obvious the dominant species of the community is.

*J* measures the species evenness; the value range is 0–1; the closer to 1, the higher the species evenness in the community.

$$H^{\prime }=-\Sigma_{i=1}^{s}PilnPi$$

Pi=Ni/N

$$D^{\prime }=N_{max}/N$$

$$J=H^{\prime }/lnN$$

where *S* denotes the number of species in the community, *N_i_* denotes the number of individuals of the i th species, *P_i_* denotes the proportion of individuals of species i to the total number of individuals, *N* represents the number of species in the community, and $N_{max}$ represents the number of dominant species.

To analyze differences in the number of pollinating insects between habitats, SPSS 27 software was used for non-parametric test analysis, and the Mann–Whitney test was used to test whether there was an overall difference. If there was an overall difference, the difference in the number of individuals between the different habitats was counted.

### 2.7. Building Pollination Networks

The pollination network relationship was investigated by recording the visit frequency of different flowering plant species and insects. A random survey was conducted to observe the flowering plants. Within 17 transects, the flowering plants were selected, and the species were recorded. A 1 m × 1 m square was drawn and observed for 6 min to record the species, number, and flower-visiting frequency of the pollinators. By observing the frequency of pollinators visiting flowers, a (P) × (A) matrix was constructed, where P represents plant species, and A represents pollinator species [19,20]. The pollination network is drawn by the bipartite package in the R language (v.4.3.3), and the pollination network structure is visualized. At the same time, the network-level function in the bipartite package of R software (v.4.3.3) was used to calculate the following pollination network parameters (connectance, links per species, network-level specialization index (H2), weighted nestedness, web asymmetry, and modules) [21,22].

### 2.8. Evaluation of Pollination Service Value

#### 2.8.1. Pollination Dependence Assessment

We referred to the list of major crops closely related to human food listed by Gallai et al. [23], combined with the data on the crop species, planting area, yield, and production cost of pollinated crops in the Hanzhong Statistical Yearbook. Then, 11 oil crops, fruits, and vegetables with large cultivation scales and closely related to human food were selected to evaluate the economic value of insect pollination.

The value of pollination was estimated using the dependence ratio method [15], whereby the value of pollination was estimated as the proportion of crop production value that could be attributed to pollination within the area.

The interdependency on pollination of pollinators was obtained through field experiments using gauze nets. The method is to select 15–20 branches or plants for pollination before flowering, count the number of flower buds, cover them with a gauze net, hang a label, and use a waterproof pen to write the net-covering time and number of flower buds on the label. After flowering and fruiting, remove the gauze net and count the number of fruit set. And select 5–7 flower branches or individuals, hang labels, but do not cover them with gauze as a contrast. The difference in fruit setting rate between branches with and without nets is known as the interdependency on pollination of pollinators. This study conducted pollination dependence experiments in Hanzhong City and the neighboring Nanchong City [10].

#### 2.8.2. Pollination Service Value

Based on the planting area and yield data of eleven pollinated crops in the 2023 Hanzhong Agricultural Statistical Yearbook, an evaluation model was constructed to calculate the value of pollination services.

The formula for calculating the total economic value (PEV) of insect pollination is as follows:

$$PEV=\sum_{i=1}^{n}\left(Di\times Qi\times Ci\right)$$

$$TEV=\sum_{i=1}^{n}\left(Qi\times Ci\right)$$

$$RV=\frac{PEV}{TEV}=\frac{\sum_{i=1}^{n}\left(Di\times Qi\times C\right)}{\sum_{i=1}^{n}Qi\times Ci}\times 100$$

In the above formula, i is the i th pollinating crop (i ∈ [1, n]), D_i_ is the dependence ratio of the crop on pollinating insects (0 ≤ Di ≤ 1), Q_i_ is the yield of the i th crop, C_i_ is the production cost, *TEV* is the total economic output value of pollinated crops, and *RV* is the ratio of the value of insect pollination services to the total economic output value of crops.

### 2.9. Data Analysis

SPSS Statistics 19 is used as a statistical analysis tool; the correlation function of Excel 2019 software was used to complete the summation calculation.

## 3. Results

### 3.1. Pollinator Diversity

A total of 59 flowering plants were recorded, and 1976 insects were collected, including 80 species, belonging to 50 genera and 13 families. Among them, 58 species of Lepidoptera, 14 species of Hymenoptera, and 8 species of Diptera accounted for 66.59%, 28.64% and 3.69% of the total species, respectively.

#### 3.1.1. Butterfly Diversity

A total of seven families, 58 species, and 1316 butterfly individuals were recorded (Table 1). Through the Shannon-Wiener diversity, Pielou evenness, and Berger–Parker dominance indexes, the significant differences in species diversity among different butterfly families were revealed.

The Shannon-Wiener diversity index (2.175) was highest for Nymphalidae, reflecting its high species richness and abundance. The Pielou evenness diversity index of Lycaenidae was the highest (0.890), indicating that the species distribution in the community was the most uniform. The number of species of Hesperidae was only three, but the Berger–Parker dominance index was as high as 0.783, indicating that they had a high abundance and occupied a dominant niche.

The butterfly resources in Hanzhong City showed a core–periphery community structure. Nymphalidae, Pieridae, and Papilionidae constitute the core of species richness and abundance. Lycaenidae and Hesperiidae play a complementary role; Species in Riodinidae and Danaidae are rare.

#### 3.1.2. Bee Diversity

A total of 579 bees were recorded. There were a total of 15 species, belonging to three families and six genera (Table 2). Of these, 562 were honeybees, accounting for 99.30%. Megachilidae accounted for 2.41%, and Vespidae accounted for 0.52% of the total. The honeybee was a dominant species.

#### 3.1.3. Diversity of Flies and Moths

A total of 73 flies were recorded, belonging to five genera. Eight *Macroglossum* sp. were recorded (Table 3).

### 3.2. Diversity of Pollinating Insects in Different Habitats

The survey showed that there were significant differences in the species, quantity, and community characteristics of pollinating insects in different habitats in Hanzhong City. The Shannon–Wiener index was highest in the agroforestry habitat; this indicates range effectiveness. The forest habitat has a more suitable survival habitat, and the agricultural habitat has rich nectar resources. The dominance index was highest in the forest habitat; it indicates some species were in large abundance (Table 4).

The results of the non-parametric test showed that there were significant differences in the effects of different habitat types on the species diversity and abundance of pollinating insects (*p* = 0.046 < 0.05). Specifically, the median estimated abundance of pollinating insects in farmland was 96.000, significantly higher than the 60.000 in the forest area (U = 3, z = −2.402, *p* = 0.016 < 0.05). This may be due to the cultivation of crops such as rape and citrus in farmland, which provides abundant food and habitat space for pollinators, but the intercropping in farmland of crops and the use of pesticides lead to low diversity.

By contrast, there were no significant differences in pollinator abundance between the agroforestry ecotone and farmland (U = 13, z = −0.8, *p* = 0.423 > 0.05), or forest area (U = 8, z = −1.607, *p* = 0.108 > 0.05), indicating that there was no obvious distinction between agroforestry ecotone and the other two habitats in the distribution of abundance (Table 5).

### 3.3. Pollinator–Plant Pollination Network

(1) Species composition of pollinating insects and flowering plants

The pollination network of Hanzhong City covered 58 pollinators and 55 flowering plants. There were 252 connections between insects and flowering plants, and 3190 connections were formed between species, accounting for 7.9% of the total number of potential connections, showing complex ecological interactions. The asymmetry of the network was 0.045, and the number of flowering plant species was less than that of pollinating insect species. Combined with the higher average number of connections of pollinating insects, this shows that the ecological service potential of pollinating insects was large and showed generalization characteristics in the network.

**Figure 3:**
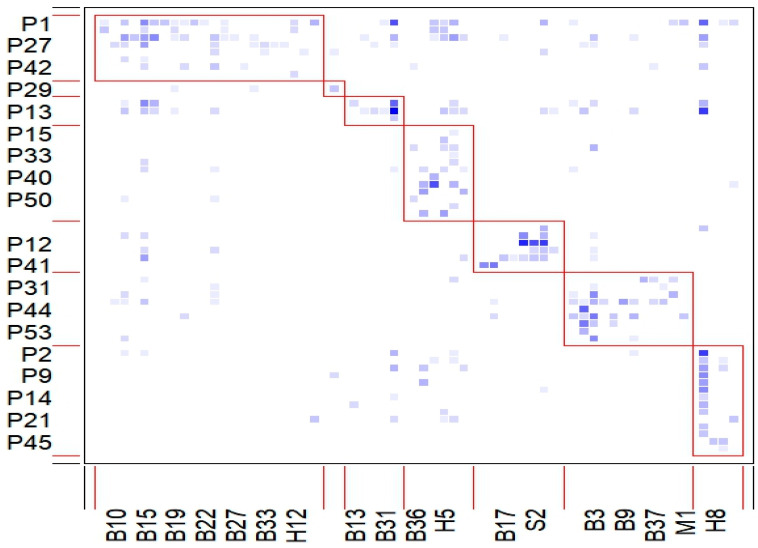
Interaction matrix featuring modules of pollination network in Hanzhong city.

In terms of network structure characteristics, the number of compartments was one, and the nested index was 0.5152, which was greater than the index of 0.4806. The weighted nestedness was also 0.5152, and the value was close to 1, indicating that the pollination network had nested structure characteristics. This can be seen from the interaction matrix in Figure 3, in which a red box identifies seven modules, representing the internal nested structure compartment; the blue area shows the interaction between species, and the color depth directly reflects the correlation strength.

In order to verify the significance of the nested structure of the network, 1000 simulation operations were performed using the bipartite installation package’s null model function in R (v.4.3.3) The results showed that *p* = 0.622 in the null model network, indicating that the network nesting index did not reach a significant level. Nevertheless, parameter analysis still shows that the pollination network in Hanzhong City presents modular characteristics, and there is a nested structure inside each module. This highly modular and low-connectivity network architecture helps maintain the stability of the community food network and is a reciprocal network that supports the stable operation of the ecosystem; see Figure 3. For data, see Appendix A, Table A1 and Table A2.

(2) Insect–plant interaction network

**Figure 4:**
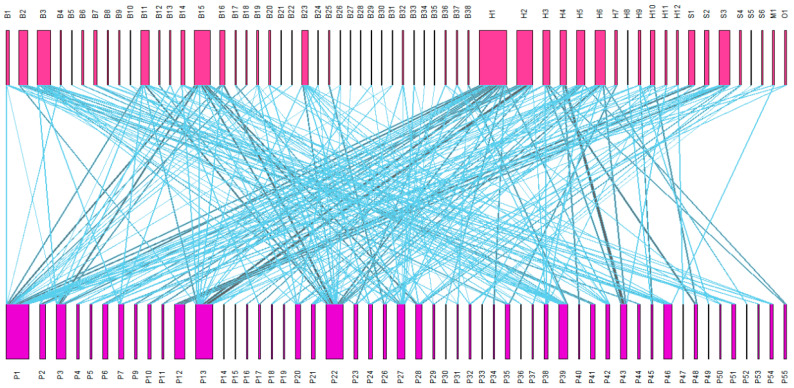
Plant–insect bipartite network diagram of Hanzhong City (The size of the rectangle corresponds to the relative abundance, the magenta rectangle represents the pollinator, and the violet-red rectangle represents the flowering plant (species code is detailed in the attached Table A1 and Table A2). The connection width between the upper and lower rectangles reflects the correlation strength).

Among the butterflies, *Pieris rapae* visited the greatest number of flowering plants (16 species), accounting for 29.1% of the total number of flowering plants. The next highest were *Papilio xuthus* and *Colias fieldii*, visiting 13 (23.6%) and 12 (21.8%) flowering plants, respectively (Figure 4).

*Apis cerana* and *Apis mellifera* were the most important bee species, which visited 18 and 14 species of flowering plants, respectively, accounting for 32.7% and 25.5% of the total plant species. They not only had a rich species of nectar plants but also had high visiting frequency (Figure 4).

The pollination effect of bumblebees was particularly significant, with the 12 species collectively visiting 42 (69.1%) flowering plant species. Flies and other insects visited 14 plant species, accounting for 25.5% of the total plant species, and play an auxiliary role in pollination.

In the pollination network of Hanzhong City, there were significant differences in pollinator diversity among different flowering plants. The data show that *Brassica napus* has 24 pollinators, accounting for 41.37% of the total number of pollinators, ranking first among all plants. *Vicia sepium* and *Trifolium repens* were visited by 20 and 17 pollinators, respectively. In addition, *Buddleja davidii*, *Zinnia elegans*, *Erigeron annuus*, *Raphanus sativus*, *Astragalus sinicus*, and other plants with longer flowering periods were visited by more than 10 pollinators.

From the analysis of species intensity, 18 of the 55 flowering plants had a species intensity greater than one; among them, the species strength of 6 crops (rapeseed, spring onion, kale, citrus, broad bean, sesame, loofah), and each crop has at least two–three pollinators, indicating that these crops can effectively use local pollinators to complete the pollination process and ensure reproduction.

The pollination network in Hanzhong City showed low connectivity and nested structure characteristics, and most flowering plants and pollinators showed a high degree of generalization. This network structure helps to maintain the stability of the community structure. It is worth noting that bee pollinators occupy an important position in the pollination network system. The development and protection of their resources are of great significance to ensuring the stability of regional ecosystems and the sustainable development of agricultural production. It is urgent to attract the attention of relevant departments and researchers.

(3) Pollination service value

Based on the gross agricultural product, yield of major pollinated crops, market price, and pollination dependence of Hanzhong City in 2023, the pollination service value (PVE) and economic contribution rate (RV) of 11 major pollinated crops in Hanzhong City were calculated and evaluated.

In 2023, the total agricultural output value of Hanzhong City was 35.2 billion CNY. The pollination service value of pollinators to these 11 major pollinated crops in 2023 was about 3524–4878 billion CNY, equivalent to 10.0–13.87% of the total agricultural output value of these crops. The greatest contribution to this value was rape (32.4–45.1 million CNY). The pollination service value of vegetable crops is about 155.8–173.2 million CNY, accounting for 4.43–4.92%; the pollination service value, and for fruit crops it was 164.2–278.1 million CNY, accounting for 4.667–7.91% of the total agricultural output value in Hanzhong City, which was the highest economic contribution rate among the three types of crops (Table 6).

## 4. Discussion

This study examined the plant–pollinator networks and the economic value of pollination services in the agriculturally important Hanzhong City region of China in order to develop strategic recommendations for local conservation and research. Our findings highlight five key messages.

(1) Hanzhong City is rich in pollinating insect resources

We identified 80 species of pollinating insects in Hanzhong City, belonging to 13 families and 50 genera, mainly butterflies, bees, and flies. The abundant pollinating insect resources provide substantial pollination services for local crops. Among them, Nymphalidae and Pieridae are the dominant families in butterflies, with high species richness. As expected, Apidae were the dominant visitor group in terms of the number of plants visited, particularly among crop species, including a number of plants that have the potential for high honey yields. This is consistent with past studies that highlight the key role of bees as pollinators in Chinese landscapes and crop pollination more broadly [16]. Although broad, our study does not cover an exhaustive census of habitats and crops, but it can be used as the basis to develop strategies for the sustainable development of agriculture.

(2) There were significant differences in the pollinator communities in different habitat types.

In common with previous studies [24], the pollinator communities differed between the three habitat types surveyed. Less species richness and aboundance in the more intensively managed systems where low plant and crop diversity dominated. By contrast, among the three habitat types surveyed, the agroforestry ecotone, in particular, was characterized by highly diverse plant and pollinator communities. This represents one of the first studies into pollinators in both Chinese agroforestry and subtropical agroforestry systems more broadly. This finding is consistent with research from temperate and tropical agroforestry systems, where the higher diversity of floral and nesting resources within these diverse systems supports more diverse plant and insect communities and greater ecosystem service provision [25,26].

(3) The insect–plant pollination network in Hanzhong City is highly nested

The pollination network in Hanzhong City displays a highly nested structure, with lower compartmentality, reflecting the asymmetry of interactions between species. Both the connectivity and the average number of species connections indicate that the network is highly generalized, meaning that the networks observed are likely to be robust to outside disturbances. Although this robustness is a positive for the stability of the ecosystem [27], the study does not cover the full plurality of habitats within the landscape, and as such, may miss some specialist plants, and network disruption that has already occurred [28]. The long-term monitoring of plants, pollinators, and their interactions would provide greater insight into the relative impacts on pollination services and wider biodiversity.

(4) The value of pollination services in the Hanzhong City region is high

In 2023, the pollination service value of 11 major pollinated crops in Hanzhong City was estimated at CNY 48.8–352.9 million, accounting for 10.02–13.87% of the City’s agricultural output value. Among them, the pollination service value of fruit and vegetable crops was higher, and the economic contribution rates were 4.67–7.90% and 4.43–4.92%, respectively. This value only represents the economic benefits to farmers and does not capture the full value of pollination services to consumers and other actors [15], which may be much higher, given value chain transformations [29]. This analysis also assumes that pollination services are already at their maximum levels, but in reality, some of this value may already be lost due to pollination service deficits, as observed in other areas globally [30]. Given the significant economic importance of agriculture to the area, monitoring and conserving pollinator populations are likely to have a significant economic benefit to the community.

(5) Recommendations for pollinator protection in Hanzhong City:

① Supporting apiculture: There are abundant nectar plants in Hanzhong City, such as rape, milk vetch, and vitex. Supporting apiculture can not only provide honey and its by-products (propolis, beeswax, royal jelly), but also increase the pollination of crops, improve food production, and promote the reproduction of wild plants. However, care should be taken to avoid excessive competition between wild pollinators and honeybees [31].
② Intercropping nectar plants: In orchards where citrus, apples, and pears are highly dependent on pollination by pollinators, planting flower plants such as *Brassica napus*, *Astragalus sinicus,* and *Vicia sepiu* can attract bees to collect honey, and having more bees is conducive to orchard pollination.
③ Promote green agriculture, accelerate the development of characteristic industries, adjust measures to local conditions, and rationally grow crops.

Strengthen existing diversified agricultural systems, such as forest gardens, family gardens, and agroforestry, which can support a more consistent resource base for pollinators. Additional recommendations are as follows: direct engagement with farmers and other interested actors to promote pollinators and pollination through scientifically or indigenous validated practices (such as crop rotation); improve the ecological infrastructure required for pollination, including semi-natural habitat patches distributed throughout productive agricultural landscapes, and provide floral resources; manage urban and recreational green spaces to increase the abundance of flowering plants that provide nectar and pollen locally; and increase the diversity and abundance of pollinators.

## Figures and Tables

**Table 1:** Diversity index of butterfly communities in Hanzhong city during 2023.

Family	Number of Species (%)	Number of Individuals (%)	Shannon–Wiener Diversity Index H′	Pielou’s Evenness Index J	Berger–Parker Dominance Index D′
Papilionidae	10 (17.24)	408 (31.00)	1.582	0.727	0.156
Pieridae	14 (24.13)	536 (40.73)	1.679	0.699	0.117
Nymphalidae	25 (43.10)	277 (21.05)	2.175	0.992	0.368
Lycaenidae	4 (6.89)	45 (3.42)	0.853	0.401	0.442
Hesperiidae	3 (5.17)	40 (3.03)	0.564	0.260	0.783
Riodinidae	1 (1.72)	7 (0.53)	0.336	0.470	0.005
Danaidae	1 (1.72)	3 (0.23)	0.338	0.770	0.002
Total	58 (100.00)	1316 (100.00)			

**Table 2:** Community composition of bees in Hanzhong City.

Family	Genus	Species	Number of Individuals (%)
Apidae	*Apis*	2	251 (43.35)
	*Xylocopa*	5	46 (7.95)
	*Bombus*	5	265 (45.77)
Megachilidae	*Mesoneura*	1	1 (0.17)
	Osmia	1	13 (2.24)
Vespidae	*Poliste*	1	3 (0.52)
Total	6	15	579 (100.00)

**Table 3:** Community composition of Syrphidae and Sphingidae in Hanzhong City.

Family	Genus	Species (%)	Number of Individuals (%)
Syrphidae	*Eristali*	1	18 (22.22)
	*Phytomia*	1	16 (19.75)
	*Syrphidae*	2	35 (43.21)
	*Syritta*	1	2 (2.47)
	*Melanostoma*	1	2 (2.47)
Sphingidae	*Macroglossum*	1	8 (9.88)
Total	6	7	81

**Table 4:** Insect composition and diversity indices in different habitats of Hanzhong City.

Habitat Type	Number of Families	Number of Genera	Number of Individuals	Abundance Index (R)	Shannon–Wiener Diversity Index (H′)	Pielou’s Evenness Index (J)	Dominance Index (D)
farmland	6	23	591	4.111	2.598	0.730	0.175
forest area	6	17	335	3.491	2.344	0.911	0.192
agroforestry	8	28	499	6.811	3.102	0.839	0.162

**Table 5:** Nonparametric test analysis results of agroforestry farmland and forest area.

	Habitat Type		Mann–Whitney Statistics U Value	Mann–Whitney Test Statistic z Value	*p*
Number of individuals	Agroforestry	Forest area	8	−1.607	0.108
	89.000	60.000			
	Agroforestry	Farmland	13	−0.8	0.432
	89.000	96.000			
	Farmland	Forest area	3	−2.402	0.016 *
	96.000	60.000			

* *p* < 0.05.

**Table 6:** Service value of insect pollination and its contribution to the agricultural output value of major crops in Hanzhong City in 2023.

Crop Common	Degree of Dependence (D)	Production (tons)	Price (CNY/kg)	Value of Services (CNY 10,000)	Economic Contribution Rate (%)
*Brassica napus*	0.28–0.39	148,400	7.80	32,410.56–45,143.28	0.921–1.283
*Malus pumila*	0.40–0.90	15,778	6.00	3786.72–8520.12	0.108–0.242
*Pyrus* spp.	0.44–0.65	60,801	5.60	14,981.3664–22,131.564	0.426–0.629
*Citrus reticulata*	0.50–0.96	458,680	3.20	73,388.8–140,906.496	2.086–4.172
*Prunus persica*	0.40–0.90	31,509	5.00	6301.8–14,179.05	0.179–0.403
*Actinidia chinensis*	0.90–1.00	68,967	8.00	49,656.24–55,173.6	1.412–1.568
*Vitis vinifera*	0.00–0.10	13,330	10.00	0–1333	0.000–0.038
*Diospyros kaki*	0.40–0.90	16,740	12.00	8035.2–18,079.2	0.228–0.514
*Prunus armeniaca*	0.63–1.00	2592	9.60	1567.6416–2488.32	0.045–0.071
*Prunus pseudocerasus*	0.43–0.63	7510	20.00	6458.6–9462.6	0.184–0.269
*Cucumis sativus*	0.90–1.00	346,440	5.00	155,898–173,220	4.431–4.924
sum			52,484.928–487,847.334		10.020–13.867

## Data Availability

The original contributions presented in this study are included in the article. Further inquiries can be directed to the corresponding author.

## Appendix A

**Table A1:** Species-level parameters of pollinators in pollination network of Hanzhong City.

Codes	Pollinators	Visit Plant Species Degree	Species Strength	Specialization Level (d′)
B1	*Papilio bianor*	6	0.40606	0.23172
B2	*Graphium sarpedon*	7	2.79802	0.68458
B3	*Papilio xuthus*	13	2.85833	0.44130
B4	*Pazala alebion*	2	0.20199	0.40646
B5	*Papilio protenor*	1	0.07142	0.35813
B6	*Byasa mencius*	1	0.46153	0.79223
B7	*Byasa impediens*	3	0.65109	0.62980
B8	*Polyura narcaea*	2	0.28205	0.47030
89	*Papilio polytes*	1	0.17857	0.54654
B10	*Papilio machaon*	2	0.12662	0.33341
B11	*Colias fieldii*	12	1.82326	0.21922
B12	*Colias erate*	1	0.05555	0.28887
B13	*Gonepteryx mahaguru*	2	0.4000	0.51180
B14	*Eurema blanda*	6	0.44819	0.24183
B15	*Pieris rapae*	16	2.77921	0.22902
B16	*Pieris canidia*	4	0.32565	0.23378
B17	*Pieris napi*	2	0.16851	0.37924
B11	*Colias fieldii*	12	1.82326	0.21922
B18	*Anthocharis cardamines*	1	0.04347	0.22856
B19	*Argyronome laodice*	5	0.37687	0.19555
B20	*Argyreus hyperbius*	4	0.28229	0.23008
B21	*Aporia tsinglingica*	1	0.04347	0.22856
B22	*Aporia crataegi*	1	0.01449	0.00000
B23	*Polygnia c-aureum*	11	1.65782	0.29989
B24	*Polygonia c-album*	2	0.03738	0.03208
B25	*Vanessa indica*	1	0.36363	0.74052
B26	*Aglais io*	3	0.09551	0.09823
B27	*Vanessa cardui*	1	0.01851	0.05789
B28	*Neptis sappho*	1	0.07692	0.39422
B29	*Coenonympha amaryllis*	2	0.08319	0.20897
B30	*Mycalesis gotama*	1	0.05000	0.29248
B31	*Aphantopus hyperanthus*	2	0.03336	0.00227
B32	*Celastrina argiolus*	3	0.26397	0.28736
B33	*Pseudozizeeria maha*	2	0.23376	0.44710
B34	*Albulina orbitula*	1	0.04545	0.26996
B35	*Lycaena phlaeas*	1	0.04545	0.26996
B36	*Erynnis montanus*	3	0.77692	0.62254
B37	*Ochlodes ochracea*	3	0.33016	0.40763
B38	*Lobocla simplex*	2	0.44047	0.49986
H1	*Apis mellifera*	18	8.74488	0.47621
H2	*Apis cerana*	11	2.84928	0.35855
H3	*Bombus atripes*	6	2.05416	0.64375
H4	*Bombus Latreille*	6	1.91616	0.58672
H5	*Bombus flavus*	10	2.77510	0.43352
H6	*Bombus breviceps*	14	4.76304	0.34039
H7	*Bombus remotus*	3	0.49651	0.45941
H8	*Xylocopa nasalis*	1	0.50000	0.82946
H9	*Xylocopa sinensis*	5	1.80615	0.57233
H10	*Xylocopa appendiculata*	6	0.84421	0.36257
H11	*Xylocopa rufipes*	3	0.35726	0.37880
H12	*Xylocopa phalothorax*	3	1.15398	0.49656

**Table A2:** Species-level parameters of plants in the pollination network of Hanzhong.

Codes	Plant Species	Visit Plant Species Degree	Species Strength	Specialization Level (d′)
P1	*Brassica napus*	24	7.80620	0.27147
P2	*Astragalus sinicus*	6	0.38164	0.23544
P3	*Raphanus sativus*	9	1.36435	0.25821
P4	*Malus sylvestris*	2	0.15650	0.29231
P5	*Prunus persica*	4	0.24017	0.18626
P6	*Cercis chinensis*	5	0.67903	0.30647
P7	*Malus spectabilis*	5	0.58366	0.39539
P8	*Paeonia suffruticosa*	0	0.00000	0.00000
P9	*Pyrus* spp.	2	0.40392	0.38345
P10	*Wisteria floribunda*	2	0.24064	0.35589
P11	*Poncirus trifoliata*	2	0.10089	0.25672
P12	*Photinia serrulata*	3	1.52483	0.74150
P13	*Trifolium repens*	17	3.65696	0.21620
P14	*Cerasus pseudocerasus*	2	0.04352	0.06664
P15	*Vicia faba*	1	0.03225	0.22704
P16	*Orychophragmus violaceus*	1	0.06000	0.30964
P17	*Rosa banksiae*	2	0.54705	0.40417
P18	*Sophora davidii*	2	0.11221	0.21890
P19	*Paulownia tomentosa*	1	0.11538	0.47010
P20	*Allium fistulosum*	7	2.04990	0.52783
P21	*Brassica oleracea*	5	1.07154	0.48876
P22	*Vicia sepiu*	20	5.51398	0.24657
P23	*Cosmos bipinnata*	4	0.64143	0.39294
P24	*Coreopsis lanceolata*	6	1.07236	0.36694
P25	*Trifolium pratense*	0	0.0000	0.0000
P26	*Rosa multiflora*	5	1.73534	0.63958
P27	*Erigeron annuus*	13	4.39642	0.36535
P28	*Ligustrum lucidum*	8	2.44347	0.43086
P29	*Cirsium arvense*	3	1.00000	0.74397
P30	*Punicagranatum*	1	0.03529	0.17943
P31	*Camassia*	3	0.32763	0.34404
P32	*Daucus carota*	5	1.66586	0.50199
P33	*Calystegia hederacea*	1	0.03225	0.22704
P34	*Melilotus albus*	1	0.03529	0.179435
P35	*Leonurus japonicus*	5	0.89374	0.48913
P36	*Verbena bonariensis*	2	0.10083	0.27018
P37	*Thladiantha dubia Bunge*	2	0.10618	0.25617
P38	*Nelumbo sp.*	7	0.47016	0.21784
P39	*Zinnia elegans*	13	4.28096	0.51146
P40	*Althaea rosea*	1	0.19047	0.58322
P41	*Clerodendrum bungei*	2	1.70000	0.94416
P42	*Rudbeckia hirta*	7	1.92055	0.38166
P43	*Lagerstroemia indica*	4	0.90600	0.57961
P44	*Cinnamomum camphora*	1	0.30769	0.67885
P45	*Luffa cylindrica*	2	1.37500	0.87119
P46	*Buddleja davidii*	8	2.53460	0.55613
P47	*Vitex negundo*	1	0.33333	0.73700
P48	*Rubus lambertianus*	2	0.53496	0.62791
P49	*Sesamum indicum*	1	0.12500	0.53193
P50	*Aster indicus*	3	0.49263	0.49757
P51	*Cayratia japonica*	3	0.74423	0.55021
P52	*Phaseolus vulgaris*	1	0.06451	0.34387
P53	*Sambucus chinensis*	1	0.07989	0.41378
P54	*Citrus reticulata*	3	0.89554	0.24017
P55	*Cosmos sulphureus*	2	0.10488	0.90087
